# TREX-2-ORC Complex of *D. melanogaster* Participates in Nuclear Export of Histone mRNA

**DOI:** 10.1134/S160767292370059X

**Published:** 2024-01-07

**Authors:** M. M. Kurshakova, Y. A. Yakusheva, S. G. Georgieva

**Affiliations:** Engelhardt Institute of Molecular Biology, Russian Academy of Sciences, Moscow, Russia

**Keywords:** TREX-2, ORC, TREX-2-ORC, Xmas-2, PCID2, ENY2, mRNA export, histone mRNA

## Abstract

The TREX-2-ORC protein complex of *D. melanogaster* is necessary for the export of the bulk of synthesized poly(A)-containing mRNA molecules from the nucleus to the cytoplasm through the nuclear pores. However, the role of this complex in the export of other types of RNA remains unknown. We have shown that TREX-2-ORC participates in the nuclear export of histone mRNAs: it associates with histone mRNPs, binds to histone H3 mRNA at the 3'-terminal part of the coding region, and participates in the export of histone mRNAs from the nucleus to the cytoplasm.

Gene expression consists of several stages: synthesis of mRNA, formation of a mature mRNP particle, and export of mRNA from the nucleus to the cytoplasm through the nuclear pores. The export receptor NXF1 is recruited to mRNA through adapter proteins, which can bind to mRNA during transcription, splicing, 3'-end formation, and intranuclear transport of mRNP to nuclear pores. NXF1 forms a heterodimer with the p15 protein (Mex67-Mtr2 heterodimer in yeast). The nuclear export receptor heterodimer mediates translocation of mRNA through the nuclear pore by interacting with FG repeat-containing nucleoporins.

One of the key regulators of mRNA export is the TREX-2 protein complex. Homologous TREX-2 complexes have been characterized in many eukaryotes, including yeast and humans. TREX-2 is capable of binding to mRNA, associates with nuclear pores, and is required for mRNA export from the nucleus in various organisms [1–7]. The TREX-2 complex of *D. melanogaster* consists of Xmas-2, PCID2, ENY2, and Sem1p proteins [8]. The Xmas-2 protein serves as a platform for the assembly of the complex, and other proteins interact with it. Previously, our team purified the joint complex consisting of TREX-2 and ORC (Origin Recognition Complex) proteins and demonstrated the role of ORC subunits in the export of mRNA [9]. The ORC complex was first described in budding yeast as a complex that binds to origins of replication and is involved in the recruitment of the Mcm2-7 complex. Later, homologous complexes were discovered in other organisms [10]. However, it was found that, in higher eukaryotes, the ORC complex and its individual subunits perform many different functions not related to replication; in particular, the interaction of ORC proteins with various RNAs was shown [11]. We showed that ORC proteins interact with the mRNP particle and that this interaction is mediated by the TREX-2 complex. ORC subunits were shown to interact with the NXF1 export receptor and are required for the binding of NXF1 to the mRNP particle. Knockdown of ORC components, as well as knockdown of TREX-2 components, leads to the disruption of poly(A)-containing RNA (mRNA) export from the nucleus. Thus, it was shown that the TREX-2-ORC protein complex of *D. melanogaster* recruits the export receptor NXF1 into mRNP particles and is a key participant in the export of the bulk of the synthesized mRNA molecules [9]. The aim of this study was to investigate the involvement of TREX-2-ORC in the export of non-polyadenylated mRNAs encoded by replication-dependent histone genes.

The mRNAs of replication-dependent histone genes (hereinafter, histone mRNAs) are synthesized at the beginning of S phase of the cell cycle and are the only eukaryotic mRNAs that do not undergo polyadenylation [12]. Histone pre-mRNAs do not contain introns; their maturation involves only capping and endonucleolytic cleavage of the 3'-end. At the 3'-end, histone pre-mRNAs contain a stem-loop structure and an HDE element, which both direct the assembly of the specific processing machinery [13]. The SLBP protein (stem-loop binding protein) binds to the stem-loop. The 5'-end of the U7 snRNA is associated with  the HDE sequence. The U7 snRNA forms the U7 snRNP together with a protein complex consisting of the Sm spliceosomal proteins, in which two proteins SmD1 and SmD2 are replaced by the Lsm10 and Lsm11 proteins. In mammals, the Lsm11 protein interacts with its extended N-terminus with the N-terminal region of the FLASH protein. The common surface of FLASH with Lsm11 recruits the HCC (histone cleavage complex) processing complex containing the factors Symplekin, CPSF73, CPSF100, CPSF160, WRD33, and Cst64 to U7 snRNP. Histone pre-mRNA is cleaved by the CPSF73 endonuclease at a distance of 4–5 nucleotides from the stem-loop. After cleavage, mature histone mRNAs are rapidly exported from the nucleus to the cytoplasm. Despite the specific structure of histone mRNAs, it was shown that they are also exported by the NXF1 receptor [14, 15]. In this work, we showed that the TREX-2-ORC complex is associated with histone mRNP particles, binds to histone H3 mRNA at the 3'-end part of the coding region, and is involved in non-polyadenylated histone mRNA export.

## *TREX-2-ORC Complex Is Associated with mRNP Histone Particles*

**Fig. 1.**
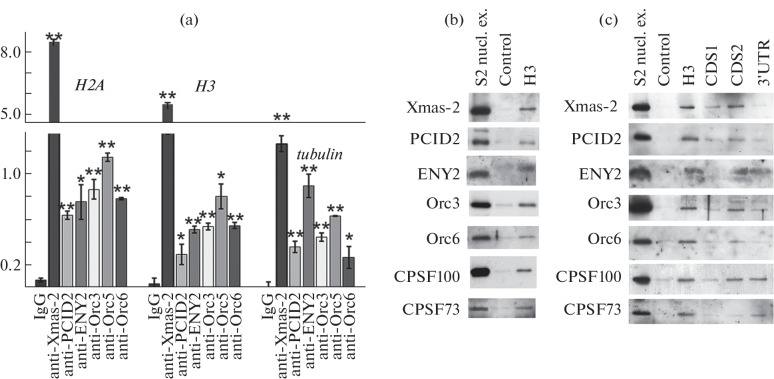
TREX-2-ORC associates with histone mRNA. (a) Results of immunoprecipitation of *H2A*, *H3*, and β-*tubulin* mRNP particles with antibodies to TREX-2-ORC subunits Xmas-2, PCID2, ENY2, Orc3, Orc5, Orc6, IgG was used as a negative control. Results are presented as a percentage of the starting material. All data are presented as mean ± standard deviation, Student’s *t*-test was used to compare control and experiment. * indicates Statistical significance at *p* < 0.05, ** indicates statistical significance at *p* < 0.01. (b) Western blot analysis of co-precipitation of *H3* mRNP particles with biotinylated antisense RNA probe to histone H3 mRNA using antibodies to Xmas-2, PCID2, ENY2, Orc3, and Orc6. RNA probe with an antisense sequence to YFP RNA was used as a negative control (Control). (c) Western blot analysis of protein coprecipitation with biotinylated antisense RNA probes to the histone H3 mRNA fragments (full-sized H3, CDS1, CDS2, and 3'UTR) using antibodies to Xmas-2, PCID2, ENY2, Orc3, and Orc6. RNA probe with an antisense sequence to YFP RNA was used as a negative control (Control).

This study was aimed to investigate the interaction of the TREX-2-ORC complex with mRNP histone particles. Previously, ORC subunits Orc1, Orc3, Orc4, Orc5, and Orc6 were found in the purified joint TREX-2-ORC complex [9]. The interaction of the complex with histone mRNPs was tested for TREX-2 subunits Xmas-2, PCID2, and ENY2 and for ORC proteins Orc3, Orc5, and Orc6. For this purpose, the method of coprecipitation of RNP particles with antibodies (RIP) from the nuclear extract of S2 D. mela-nogaster cells was used. Antibodies to the TREX-2 and ORC protein components effectively precipitated the mRNA of the histone genes *H2A* and *H3* (Fig. 1a), indicating the interaction of the TREX-2-ORC complex with mRNP particles of these histones. Similar data were obtained for *H4* and *H2B* mRNA. The interaction of TREX-2-ORC with poly(A)-containing mRNA of β-tubulin gene, which was demonstrated previously [9], served as a positive control.

The interaction was further confirmed using an alternative approach: mRNP particles containing the *H3* gene transcript were isolated from the nuclear extract of S2 cells using a biotinylated antisense RNA probe to histone *H3* mRNA (Fig. 1b). The antisense RNA probe specifically associates with the corresponding endogenous RNA in the extract, precipitating the RNP particle. The mRNP particles bound to the RNA probe were precipitated from the extract using streptavidin-agarose, and mRNP proteins were analyzed using Western blot analysis with antibodies to the TREX-2 and ORC components. Precipitation with an antisense sequence to YFP RNA served as a negative control. Subunits of the TREX-2 and ORC complexes, proteins Xmas-2, PCID2, ENY2, Orc3, and Orc6, as well as processing factors CPSF73 and CPSF100 coprecipitated with *H3* mRNP but were absent in the negative control. Thus, the TREX-2-ORC complex is able to associate with histone mRNP particles.

## *TREX-2-ORC Associates with H3 mRNA at the 3'-End Part of the Coding Region*

To localize the region of *H3* mRNA where TREX-2-ORC is associated, biotinylated sense RNA probes corresponding to various regions of the *H3* RNA sequence were incubated with the nuclear extract of S2 cells, and the proteins bound to the RNA probes were detected. The probes corresponded to the full-length (H3), N- and C-terminal regions of the 411-nt coding region (CDS1: 1–204 nt and CDS2: 204–411 nt, respectively) and the 3'-untranslated region of the H3 pre-mRNA (3'UTR: 412–595 nt) (Fig. 1c). The Xmas-2, PCID2, Orc3, and Orc6 proteins associated predominantly with the CDS2 sequence. The processing factors CPSF73 and CPSF100, used as control factors, were strongly associated with the 3'UTR probe, which contains a hairpin structure and the HDE element, directing the assembly of the processing apparatus; this confirms the specificity of the identified interactions. ENY2 coprecipitated with both CDS2 and 3'UTR RNA probes, which can be explained by the ability of ENY2 to be recruited into various protein complexes. The proteins did not interact with the control RNA probe. Thus, the TREX-2-ORC complex proteins associate with the coding sequence of histone *H3* RNA at the 3'-end part of the coding region.

## *TREX-2-ORC Is Involved in Histone mRNA Export*

Next, we studied the involvement of TREX-2-ORC in the export of histone mRNA from the nucleus to the cytoplasm. The Xmas-2 protein is a platform with which the other proteins of the TREX-2-ORC complex are associated: PCID2 is associated with the Xmas-2 region near the GANP domain, EYN2 is associated with the CID domain of Xmas-2. Of all ORC subunits of the complex, Orc3 binds most strongly to Xmas-2, interacting together with ENY2 with the C-terminus of Xmas-2 [16, 17].

**Fig. 2.**
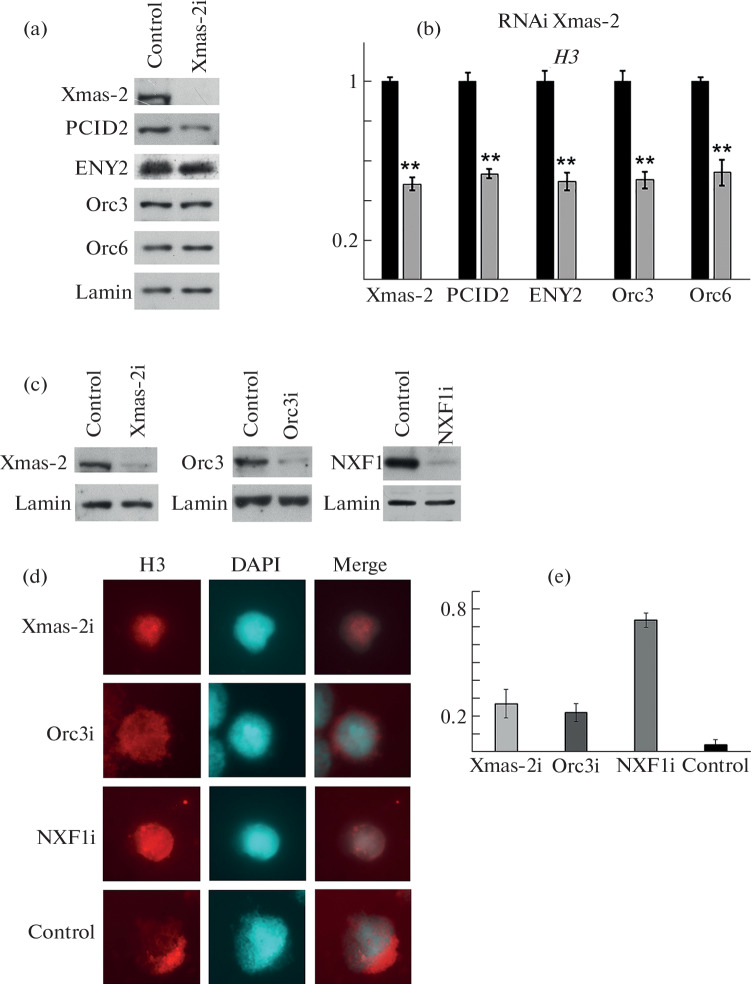
Effect of knockdown of TREX-2-ORC subunits on histone mRNA export. (a) Western blot analysis of protein levels after knockdown of Xmas-2, in S2 cells using the corresponding antibodies. Staining with antibody to lamin Dm0 was used as an alignment control. (b) Results of the immunoprecipitation of histone mRNPs after Xmas-2 knockdown with antibodies to TREX-2-ORC subunits, using IgG as a negative control. Results are presented as a percentage of the starting material. All data are presented as mean ± standard deviation, Student’s *t* -test was used to compare control and experiment. * indicates Statistical significance at *p* < 0.05, ** indicates statistical significance at *p* < 0.01. (c) Western blot analysis of Xmas-2, Orc3, and NXF1 knockdown protein levels using antibodies to corresponding proteins. Staining with antibodies to lamin Dm0 was used as an alignment control. (d) FISH hybridization with a DIG-labeled antisense RNA probe to *H3* mRNA after knockdown of TREX-2-ORC components Xmas-2, Orc3 and NXF1 (Xmas-2i, Orc3i, NXF1i) in S2 cells. The effect of GFP protein knockdown is shown as a negative control. (e) Proportion of S2 cells with impaired mRNA export after the knockdowns of Xmas-2, Orc3, NXF1 or GFP (control).

Therefore, to reduce the level of the TREX-2-ORC complex, Xmas-2 in S2 cells was knocked down using RNA interference. The level of Xmas-2 expression significantly decreased, whereas the expression levels of the PCID2, ENY2, Orc3, and Orc6 subunits did not change noticeably (Fig. 2a). Using RIP analysis, we tested whether Xmas-2 knockdown disrupts the interaction of TREX-2-ORC with histone mRNPs. Xmas-2 knockdown led to a decrease in the amount of both Xmas-2 and the PCID2, ENY2, Orc3, and Orc6 proteins associated with histone mRNAs, that is, to a decrease in the binding of TREX-2-ORC to mRNP particles of histones. The results are shown for *H3* mRNA (Fig. 2b).

Next, we studied how the disruption of the interaction of histone *H3* mRNA with TREX-2-ORC complex affects its export from the nucleus to the cytoplasm. To do this, we analyzed the distribution of *H3* mRNA in S2 cells upon knockdown of Xmas-2 and Orc3 (the key subunits for for the interaction of complex with mRNP and NXF1) using fluorescent in situ hybridization (FISH) with a DIG-labeled antisense RNA probe to the coding sequence of the *H3* gene, stained by anti-DIG rhodamine. (Figs. 2d, 2e). Since histone mRNA is synthesized during the S phase and undergoes degradation after the end of the S phase, the FISH signal corresponding to H3 mRNA was detected only in some cells. In the control experiment, the FISH signal of *H3* mRNA was detected mainly in the cytoplasm of the cells. Knockdown of NXF1 resulted in retention of *H3* mRNA in the nucleus, as was shown in the literature [15]. Knockdown of Xmas-2 and knockdown of Orc3 also led to disturbances in mRNA export in part of the cells with the *H3* FISH signal (about 20–30%), namely, to accumulation of RNA in the nucleus or uniform distribution between the nucleus and the cytoplasm (Figs. 2d, 2d). Thus, a decrease in the amount of TREX-2-ORC bound to mRNP particles of histones leads to disruption of the nuclear export of their mRNA. The fact that export disruption is observed only in some cells can be explained by the fact that, in addition to the TREX-2-ORC complex, other adapters (SR proteins 9G8 and SRp20 [18] and the ALYREF protein [19]) are involved in the recruitment of NXF1 to histone mRNA.

We have previously showed that the TREX-2-ORC complex associates with the major export receptor NXF1 and serves as an adapter for the binding of NXF1 to poly(A)-containing mRNAs [9]. Histone mRNAs are exported by NXF1 [15]. We have shown that the TREX-2-ORC complex is part of the mRNP particle and binds to non-polyadenylated histone mRNAs. The fact that knockdown of the complex-forming subunits of TREX-2-ORC leads to the disruption of the binding of the complex to histone mRNAs and disruption of their export in some part of S2 cells suggests that TREX-2-ORC also serves as an adapter for NXF1-dependent export of non-polyadenylated mRNAs histones, possibly participating in export at a certain stage of expression regulation during the cell cycle.

Previously, several adapters were discovered through interaction with which the NXF1 export receptor is recruited to histone mRNA. In mammalian cells and Xenopus oocytes cells, the SR proteins 9G8 and SRp20 were shown to bind to a specific transport *cis*-element at the mouse *H2a* coding region [18]. In human cells, the major export adapter ALYREF binds to the histone mRNA at a region located upstream of the cleavage site, with the peak at 50 nt [19]. According to our data, TREX-2-ORC also preferentially binds to histone mRNA at the 3'-terminal part of the coding region. However, while the binding of ALYREF to histone mRNA occurs cotranscriptionally and is associated with the landing of the processing apparatus [19], TREX-2-ORC can be recruited to histone mRNP particles at a later stage of the mRNP export pathway in the nucleus from transcription sites to nuclear pores rather than during transcription. This can be inferred from the data that knockdown of TREX-2 components does not affect the processing of poly(A)-containing model mRNAs [20].
